# Supplementary material for: Comprehensive Response of Rhodosporidium kratochvilovae to Glucose Starvation: A Transcriptomics-Based Analysis
Source: Microorganisms. 2023 Aug 27;11(9):2168. doi: 10.3390/microorganisms11092168 (PMC10534369; doi:10.3390/microorganisms11092168)
Supplement: Supplementary file 1 [file microorganisms-11-02168-s001.zip › Supplementary Figures.pdf]

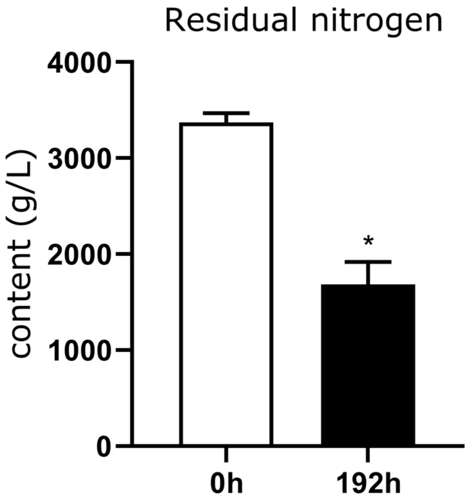

Figure S1. The residual amount of total nitrogen. Data are presented as mean ± standard deviation of three independent experiments. Statistically significant differences are indicated (* $p < 0.05$).

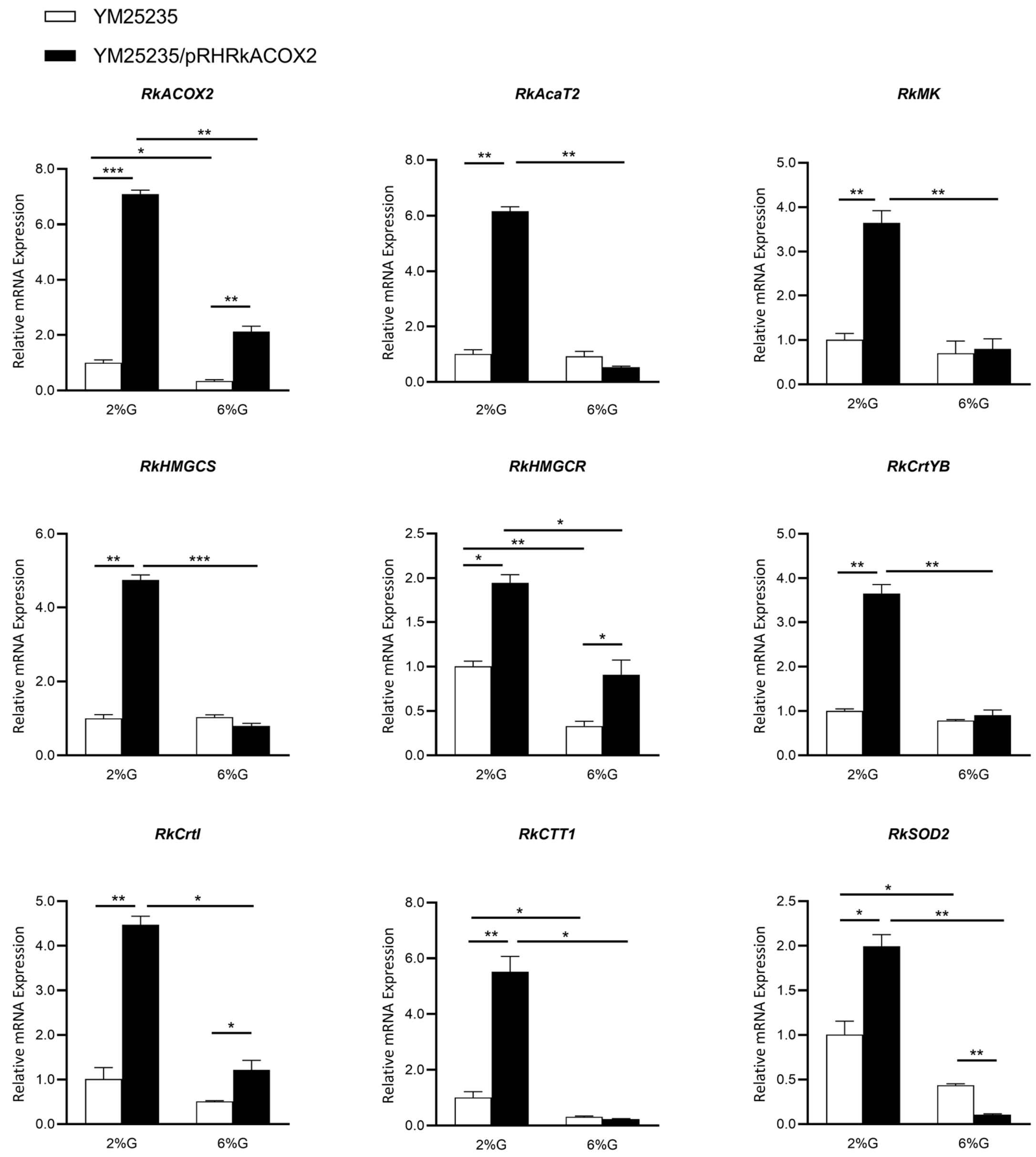

Figure S2. qPCR analysis under glucose presence and glucose starvation conditions. 2% G: YPD contains 1% yeast extract, 2% peptone, and 2% glucose; 6%G: YPD contains 1% yeast extract, 2% peptone, and 6% glucose (* $p < 0.05$, ** $p < 0.01$, *** $p < 0.001$).

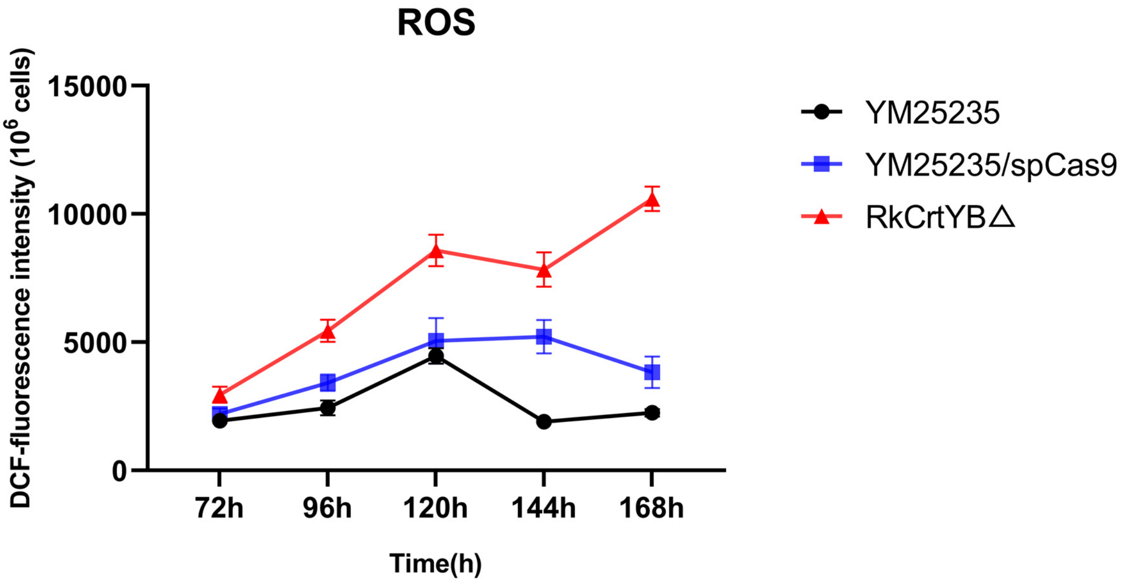

Figure S3. The ROS levels in the three YM25235 strains under glucose starvation.

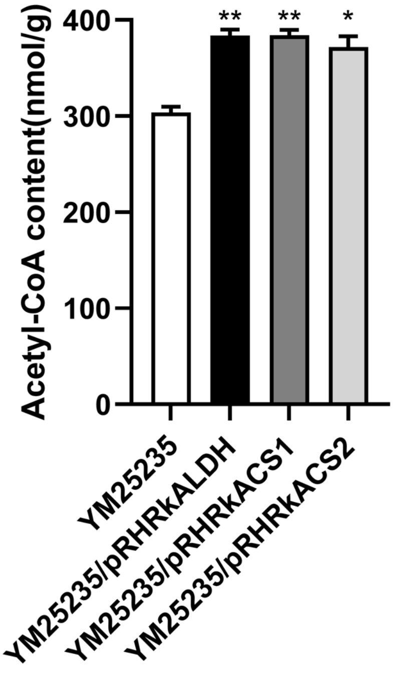

Figure S4. The acetyl-CoA level in the four YM25235 strains under glucose starvation. Data are presented as mean ± standard deviation of three independent experiments. Statistically significant differences are indicated (* $p < 0.05$, ** $p < 0.01$).
