# Supplementary material for: Comprehensive Response of Rhodosporidium kratochvilovae to Glucose Starvation: A Transcriptomics-Based Analysis
Source: Microorganisms. 2023 Aug 27;11(9):2168. doi: 10.3390/microorganisms11092168 (PMC10534369; doi:10.3390/microorganisms11092168)
Supplement: Supplementary file 1 [file microorganisms-11-02168-s001.zip › Table S1. Primer pairs used for quantitative real-time PCR.pdf]

Table S1. Primer pairs used for quantitative real-time PCR.

Gene	Primer sequence (5'-3')	
SSU rRNA	F: CCATTCACTTACAAACACAA	R: CACCACCAGATTCACTAA
BADH	F: ATCTACAACTTCCTCTTCTGG	R: CGGATGATGGACGTGAAG
FAE2	F: CTCTTCGTTCATGTGTTG	R: GTCGTGAGGTACTTCTTC
Adh	F: GTCAAGGACTGGATCAACAA	R: TCAGGCGTCGTCTTGTAG
UGDH	F: ATCCTTAACCTCGTCTAC	R: TTCTGGTACTCGTTCATC
RTK	F: ACCAACGAACAGCAGATG	R: AAGAAGACCATGACAGCAA
Git3	F: GAGTCTGGGAGGGTACGTCT	R: CGCAGAGCAAGCTGATGAAC
GSS5	F: TTCAACCTGCCACCCGTATC	R: GCGGAGGTACGAGTAGTGTG
AOX	F: AAGGAGGAGTCGTTCAAG	R: CTCGTTCTGGTAGTTGTTC
GCH2	F: CTCGGCGTAAAGAAGATC	R: TCTCATACCTGCGAACTA
ICL	F: GACGAGAACCAGATGAAGAG	R: GGCGAGGGTGATGAACTG
ppg4	F: AACAACTACTTTCAAGGG	R: CTTCATCATGCTCTTCCA
SNF1	F: ACCTCTGTGGCAAGATCAGC	R: AGAAGCTCTGAATCGCCTCG
ACOX2	F: ATTCACGACCTCACCAAGGC	R: CACTCCATATCGCGTCCAGA
ACAA1	F: GTCGAGTCGATGACCCAAGG	R: ACGTTCTCGCTCGTGATACC
NRG	F: GGGTCTACTCGAAGTGTCGG	R: TCTTGAGGTCTTCCCACTCG
AcaT2	F: CCGAACATTATGGGCATC	R: CGATGTCCTCAATCTTCAT
HMGCS	F: TTCCTCCTCTCCGTGACCAA	R: CGTCTTGACCGACTTGCTCT
HMGCR	F: CATTGTCACCGTCTTCTG	R: GTCGTCGTAGAGGAGGAA
MK	F: ACACGATCAACCCGCTCTTC	R: GAGGGACGAAAGCTCAACGA
CrtYB	F: GTTCTTCTTGTGGGAGTG	R: CCGCTTCTTCAATCTCAA
CrtI	F: GAGAAGGGTTTCGAGGGCTT	R: ATGGAGAGGAGCGAGGTGAA
CTT1	F: CAGGTCATCCCCGACAACTC	R: GAGGGTCTTTGAGCGGTTGA
SOD2	F: CGCATGTCCTGACTCTCTCC	R: AGGTTCTCCCAGAAGAGCGA
