# Supplementary material for: Comprehensive Response of Rhodosporidium kratochvilovae to Glucose Starvation: A Transcriptomics-Based Analysis
Source: Microorganisms. 2023 Aug 27;11(9):2168. doi: 10.3390/microorganisms11092168 (PMC10534369; doi:10.3390/microorganisms11092168)
Supplement: Supplementary file 1 [file microorganisms-11-02168-s001.zip › Table S4. Total lipid analysis under glucose starvation.pdf]

Table S4. Total lipid analysis under glucose starvation.

Strain	Total lipid (mg/g DCW)
YM25235	38.38±0.26
YM25235/pRHRkACOX2	39.01±0.51
