# Supplementary material for: Comprehensive Response of Rhodosporidium kratochvilovae to Glucose Starvation: A Transcriptomics-Based Analysis
Source: Microorganisms. 2023 Aug 27;11(9):2168. doi: 10.3390/microorganisms11092168 (PMC10534369; doi:10.3390/microorganisms11092168)
Supplement: Supplementary file 1 [file microorganisms-11-02168-s001.zip › Table S5. Analysis of carotenoid content under glucose presence and glucose starvation conditions..pdf]

Table S5. Analysis of carotenoid content under glucose presence and glucose starvation conditions.

Strain	Glucose	Total carotenoid（mg/g(DCW)）
YM25235	2%	5.68±0.23
	6%	2.43±0.12
YM25235/pRHRKACOX2	2%	7.59±0.35*
	6%	2.35±0.05

2%G: YPD contains 1% yeast extract, 2% peptone, and 2% glucose; 6%G: YPD contains 1% yeast extract, 2% peptone, and 6% glucose. Data are presented as mean±standard deviation of three in-dependent experiments. Statistically significant differences are indicated (*$p<0.05$).
